# Digitally Savvy at the Home Office: Computer Skills of Older Workers During the COVID-19 Pandemic Across Europe

**DOI:** 10.3389/fsoc.2022.858052

**Published:** 2022-04-26

**Authors:** Ronny König, Alexander Seifert

**Affiliations:** ^1^Department of Sociology, University of Zurich, Zurich, Switzerland; ^2^Institute for Integration and Participation, School of Social Work, University of Applied Sciences and Arts Northwestern Switzerland, Olten, Switzerland

**Keywords:** home office, corona, COVID-19, digital skills, Europe, older adults, SHARE

## Abstract

Digital skills can be a valuable resource in work life, especially in such times as the current COVID-19 pandemic, during which working from home has become new reality. Although increasing numbers of older employees (aged 50 years and above) are using digital technologies to work remotely, many of these older adults still have generally lower digital skills. Whether the pandemic will be a push factor for the acquisition of computer skills in late working life remains unclear. This study investigated the explanatory factors of the computer skills gained by older workers who were working from home during the COVID-19 pandemic, using representative data for 28 countries from the Survey of Health, Aging and Retirement in Europe (SHARE). The analysis of the survey responses of 11,042 employed persons aged 50 years and older revealed that, 13% worked only at home due to the pandemic, while 15% said they worked at home and in their usual workplace. The descriptives indicate that full-time homeworking is more of an option among those with tertiary education and who already have some computer skills. Of the older employees who worked only at home, 36% reported an improvement in their computer skills, whereas of the older workers who worked at home and at their usual workplaces, only 29% reported such an improvement. Our results based on logistic regressions suggest that significantly more women, younger employees, respondents with tertiary educational qualifications, and those whose work was not affected by unemployment or even business closure acquired new computer skills, regardless of whether they were working permanently or only partly from home. The study underlines the importance of investigating the possible digital skills gained from the home office situation resulting from the pandemic.

## Introduction

The digitalization of society yields opportunities to maintain daily life activities (e.g., online classes, shopping, medical consultation) and stay engaged with social ties, even in the midst of a pandemic. However, for individuals who are not adept at using technology or have difficulty adapting to technological innovations, living in a digitalized society may be problematic (Lupton, 2015). Further, many people in later adulthood do not have direct or immediate access to such new technologies and, thus, have fewer opportunities to use these devices to garner additional benefits for their daily working lives (Francis et al., 2019; Niesel and Nili, 2021). Many older adults, who did not grow up with today's technologies (e.g., the internet and video conferencing), seldom use the latest information and communication technologies (ICTs), unlike younger age groups (Cotten, 2021). This perceived gap between people who have access to ICTs and those who do not or cannot access ICTs is referred to globally as the “digital divide” (Compaine, 2001).

On the basis of the life course paradigm, Sackmann and Winkler (2013) presented the following technical generations: (1) the mechanical generation (born before 1939), (2) the generation of the household revolution (born 1939–1948), (3) the generation of technology spread (born 1949–1963), (4) the computer generation (born 1964–1978), and (5) the internet generation (born after 1978). This concept of different cohort-specific relationships with technology illustrates the importance of ICT exposure as a function of historical timing and technology proliferation. This historical timing and life stage influence the types of ICTs used by different age cohorts. Furthermore, many individuals in these cohorts have not encountered ICTs in their workplaces. For example, historically, to conduct meetings, they may have relied extensively on face-to-face meetings rather than online video conferencing tools.

With the internet, social interactions over a long distance have become possible; for instance, video chatting via the internet can be useful for visual interactions when geographic distance or health limitations prevent in-person interactions. For older adults, the use of the internet allows them to find useful information, access health information, and connect with others via online communities and social media (Leist, 2013; Cotten, 2021). Although internet use has been increasing among older adults, a digital gap remains between age groups (Pew Research Center, 2021). For example, a representative survey conducted across European countries showed that only 49% of people aged 50 years and older used the internet; nevertheless, a divide exists between northwest European countries (e.g., 83% of Denmark's population uses the internet) and southeast European countries (e.g., only 27% of Croatia's population uses the internet) for this group of adults (König et al., 2018). In this study, internet use among older adults was influenced by personal factors, such as age, gender, education, and income. Participants aged 80 years and older reported spending less time online than those aged 65–79 years. Further, men and older adults with higher educational and economic profiles were more likely to use the internet. Health, prior experience with technology, social salience (i.e., internet use among members of one's social network), and the communication technology infrastructure of the country of residence were also predictors of internet use by older adults (König et al., 2018). Another study using recent data from 13 European countries showed that 53% of people aged 50 years and older use the internet; however, health limitations (e.g., subjective health and grip strength) were identified as important factors that affect older adults' ability to remain online (König and Seifert, 2020). Moreover, both studies highlight differences in ICT usage based on employment status, whereas compared to retirees, employees were significantly more likely to use the internet recently (König et al., 2018). Leaving the labor force and entering retirement also marks a change in individual internet use, since new pensioners were more likely to stop using the internet and thus become more often so-called “offliners” (König and Seifert, 2020).

With the digitalization of work advancing rapidly, daily work has been increasingly more dependent on employees' use of various types of digital technologies (Warhust and Hunt, 2019). Today, for workers, investing in computer skills has become more important. Theoretically, this can be framed with the concept of “digital capital” (Park, 2017; Ragnedda, 2018). In this vein, digital capital is a recent concept that advances the understanding of digital inequalities by conceptualizing the set of abilities, aptitudes, and external resources that enable individual digital engagement and its related gains (Ragnedda et al., 2020). Digital capital refers to the accumulation of digital competence and access throughout a person's life. While digital competence refers to one's acquired and owned abilities to operate digital technologies, digital access covers the external resources available to individuals for full electronic participation. These two components are fluid and susceptible to development (e.g., through training and enhanced availability of devices). Digital capital varies greatly among groups of individuals in terms of, for example, age, education, income, and place of residence (Ragnedda et al., 2020). As described earlier, workers aged 50 years and older in particular have less internet experience (König et al., 2018; Pew Research Center, 2021) and fewer digital skills (Anderson et al., 2019; Francis et al., 2019; Cotten, 2021) and were therefore more likely to be confronted with a digital capital gap when they needed to work remotely at a digitally dominated home office.

The current COVID-19 pandemic reminds us of the significance and persistence of the digital divide and fosters discussion of the positive and negative outcomes of using vs. not using technologies (e.g., the internet) during a time of physical distancing (Xie et al., 2020). When physical distancing mandates started being implemented at the start of the COVID-19 pandemic, older adults, one of the most at-risk groups for COVID-19, were prevented from physically interacting with their social ties in person and were told to refrain from going out to work (if possible) or from visiting stores, restaurants, and other establishments (Ayalon et al., 2020). However, older adults who had access to and the ability to use ICTs could still maintain contact with their social ties, purchase food and groceries, and stay engaged in working life via their home office. By contrast, older adults who did not use ICTs, in addition to having possible age-related health and/or mobility limitations, were likely to struggle with a double burden of social exclusion; that is, older adults with fewer digital skills were not only excluded from physical contact but were also unable to compensate for face-to-face social interactions with digital solutions, such as video chatting and online social media (Robinson et al., 2020; Seifert et al., 2021). This struggle with online participation influences older adults' access to online services and content, such as social events and social networking, during a time when digital solutions can compensate for missing physical contacts (Marston et al., 2020).

Health authorities and governments worldwide have categorized older adults as a “risk group” for more serious and possibly fatal illnesses associated with the current coronavirus (COVID-19) infection (Brooke and Jackson, 2020). Consequently, measures requiring older adults to shelter in place and maintain physical distancing from others have been enacted in many countries during the pandemic. Older workers (aged 50 years and above) and employees with preexisting conditions were therefore encouraged to work from home, if possible (Engstler et al., 2020). Working from home, however, requires additional technical prerequisites in the home, such as the hardware (e.g., personal computer) and software (e.g., video conferencing application) needed to set up the home office and the digital skills needed for the digital solutions necessary for working at home (Milasi et al., 2021).

Reinforced by the COVID-19 pandemic, access to ICTs and skills in using them have become critical to participation in society, as well as in working life, when a home office is recommended by employers or mandated by the government. Contemporary work life is characterized by vast and rapid digitalization, that is, an increase in the use of digital technology by industry and companies for administration, service delivery, and production (Warhust and Hunt, 2019; OECD, 2020a) and, thus, by workers who have to face new technical challenges and learn computer skills even in their later professional careers (van Laar et al., 2020). This is manifested, for example, in many companies expanding their uptake of digital technologies, adopting new modes of production for existing or new products, moving or enlarging their businesses online, partially or completely converting to digital service delivery, and supporting their employees with new digital equipment for working on-site or outside company premises. The COVID-19 pandemic has forced an acceleration of such digital transformation of work, with companies mobilizing more resources for the digital shift and workers being exposed to more digital work (Nagel, 2020; Sostero et al., 2020). These new challenges for digitally supported work (at home) also affect the training of older people in the form of continuing education. In this context, education has been an important strategy for combating digital inequality and improving the labor market opportunities of older adults (Garcia et al., 2021).

## Research Questions

Based on the partial fragility of the digital capital of older workers (≥ 50 years of age), discussed above, and how this fragility has been exacerbated by the current COVID-19 pandemic, we investigated the intensity and the explanatory factors of the computer skills gained from home office work during the COVID-19 pandemic in a large representative dataset of older adults aged 50 years and older in Europe. Therefore, the study addresses the following research questions: (a) Did the computer skills of older workers change during pandemic-related homeworking? (b) If so, who among the older workers was able to acquire new computer skills?

## Data and Methods

### Data

Our analyses are based on the Survey of Health, Aging, and Retirement (SHARE), which provides standardized information on respondents aged 50 years and older in various European countries. The main sample employed in this study was gathered from the first COVID-19 survey as part of the eighth (2020) SHARE wave, covering 28 European countries (including Israel) [for details on the data used, see Börsch-Supan (2022e)]. These specific data on 57,559 respondents were collected via computer-assisted telephone interviews (CATIs), mainly between May and September 2020, and covered a wide range of important life domains and changes caused by the global spread of COVID-19. The countries that participated in this wave were Austria, Belgium, Bulgaria, Croatia, Cyprus, the Czech Republic, Denmark, Estonia, Finland, France, Germany, Greece, Hungary, Israel, Italy, Latvia, Lithuania, Luxembourg, Malta, the Netherlands, Poland, Portugal, Romania, Slovakia, Slovenia, Spain, Sweden, and Switzerland.

For the purpose of this study, the respondents were selected in a two-stage process. First, the initial sample covering the overall situation of employed Europeans included 11,944 respondents who were employed or self-employed (including those working in family businesses) when COVID-19 broke out (*n* = 45,524 not employed such as retired or unemployed, and *n* = 90 no information). Out of this number, we further excluded those whose daily work did not require them to use a computer (*n* = 135) or the internet (*n* = 65). Moreover, we had to exclude respondents below the target age of SHARE and, thus, those respondents who were younger than 50 years (*n* = 184), those living in nursing homes (*n* = 24), and those with a missing value in one of the explanatory variables (see below) used in our multivariate setting (*n* = 494). Considering these exclusions, the first sample included 11,042 valid interviews of employed and self-employed Europeans (see Table 1 for a descriptive overview).

**Table 1:** Characteristics.

**Parameters**	**Min**	**Max**	**Total**	**Worked at the usual workplace**	**Worked from home and the usual workplace**	**Worked at home only**	**None of these**
**Computer skills**							
Excellent			0.08	0.06	0.10	0.17	0.04
Very good			0.15	0.11	0.32	0.22	0.09
Good			0.28	0.26	0.33	0.35	0.26
Fair			0.23	0.23	0.18	0.15	0.30
Poor/None			0.18	0.23	0.02	0.04	0.27
Missing			0.08	0.10	0.05	0.07	0.04
Female	0	1	0.46	0.44	0.46	0.53	0.46
Year of birth	1929	1969	1960	1961	1961	1960	1960
Tertiary education	0	1	0.31	0.21	0.57	0.62	0.17
Migrant	0	1	0.07	0.07	0.06	0.06	0.07
Living alone	0	1	0.19	0.17	0.20	0.20	0.21
Affected by unemployed, laid off or business closure	0	1	0.20	0.11	0.15	0.12	0.58
Total			1.00	0.54	0.15	0.12	0.19
*N*			11,042	5,913	1,473	1,724	1,932
Included in further analysis					✓	✓	

*Data sources: Survey of Health, Aging and Retirement in Europe (SHARE), wave 8, COVID-19 Survey 1, release 8.0.0, weighted, own calculations*.

Based on the respondents' employment situations at the time of the survey, we were able to distinguish four distinct places of work among them: (1) their usual workplace only (*n* = 5,913); (2) their home and, alternately, their usual workplace (*n* = 1,473); (3) their home only (*n* = 1,724); and (4) neither their home nor their usual workplace (*n* = 1,932), as they lost their job or closed their businesses due to the outbreak. Since only employed respondents who worked partially or entirely at home due to the outbreak of COVID-19 were further asked, “Did you learn new computer skills?,” we analyzed the growth of individual computer skills of older workers working from home due to the pandemic by restricting our multivariate sample to those 3,197 individuals.

### Dependent Variable

The dependent variable was information on the respondents' acquired computer knowledge due to their home office-related work during the pandemic. The respondents who mentioned working at home partially or entirely during the pandemic were asked, “Did you learn new computer skills?” They could choose to answer “No” (coded as “0”) or “Yes” (coded as “1”).

### Independent Variables

Several variables covering the respondents' demographics, as well as their working and living conditions during the pandemic, were included in the empirical models to investigate the explanatory factors for computer skill gain.

As our sample focused on respondents who had to set up a home office as an economic consequence of the COVID-19 crisis, our analysis differentiated between those who worked entirely at home and those who worked both from home and in their usual workplace. Further, we included information on how many of the respondents experienced reduced working hours, increased working hours, or the same working hours since the outbreak of the pandemic. We further included information on whether respondents were affected by unemployment (such as being laid off), and even information on business closures resulting from the pandemic.

As the work of all the respondents required the use of ICT, they were further asked, “Was your internet connection adequate?” Again, their answer options were “Yes” or “No.” Since we were investigating the question of whether working from home led to an improvement in computer skills, we fell back on the individual level from previous surveys. In detail, since the fifth wave of SHARE, all the respondents were able to rate their computer skills on a five-point Likert scale that ranged from “Excellent” to “Poor,” with one response added for spontaneous answers like “I never used a computer,” which we added to the category “Poor.” If a respondent answered this question in more than one wave, we used the more recent information. As not all respondents who participated in the first SHARE COVID-19 survey were asked this question or did not answer in a previous wave, we added a category for those respondents that we labeled “Missing.”

Since the use of modern ICT often requires corresponding cognitive skills, we also included respondents' cognitive skills using the last available value from the first trial of the so-called ‘Ten words list learning test' from previous waves. In that test, the respondents were shown a list of 10 words. They were then asked to recall as many words as possible, resulting in a score ranging from 0 to 10. Moreover, as physical and psychological health plays an important role in the opportunities to acquire or deepen individual knowledge, especially during a pandemic, we considered the respondents' health conditions as well as their personal traits. Regarding their health condition, respondents were asked, “Before the outbreak of COVID-19, would you say your health was excellent, very good, good, fair, or poor?” Due to the comparatively low occurrence of “poor” health responses, we added “Fair” to the responses.

As the pandemic has had long-lasting effects on everyone's daily lives, including feelings of fear, insecurity, and powerlessness, we also considered psychological and personality-related questions that covered some possible accompanying symptoms and prospects. These questions first referred to situations in which the respondents recently felt “nervous, anxious, or on edge” and accounted for changes since the outbreak of COVID-19 by further asking those who answered “Yes” if such symptoms had “increased” since the outbreak of COVID-19. Especially during a pandemic, past and present situations can not only impact the psychological conditions of individuals but also their views and expectations of the future. Such changes can reflect their personality, which, in turn, can affect their current behavior and actions, as well as their motivation and willingness to deal with new technical requirements in a new working environment (their own homes). Therefore, we included two further questions to indicate the respondents' degree of positivity: “What was your most uplifting experience since the outbreak of COVID-19, or in other words, what experience did you have that made you hopeful or happy?” and “What are you looking forward to doing most once COVID-19 abates?” Both questions had three answer categories: some respondents did not name anything, others named something right away, and others hesitated before they named something.

Finally, we controlled for the following sociodemographic data of the respondents: their year of birth, sex (0 = male and 1 = female), and their highest educational attainment. Based on the respondents' level of education according to the International Standard Classification of Education (ISCED), we included information whether respondents held a tertiary educational qualification (ISCED 5 and higher) or not. Moreover, we considered the cultural and occupational differences caused by migration, in which case we qualified migrants as not born in their country of residence. In addition to individual characteristics, we controlled for the situation in which the respondent lived alone (“No” = 0 and “Yes” = 1).

To consider national differences regarding ICT infrastructure, we included the general distribution of broadband internet access in each of the 28 countries. Based on the national share of households with internet broadband access (in % of total households), we computed three distinctive categories of internet access across all our included countries. We labeled seven countries (Austria, Belgium, Czech Republic, Hungary, Italy, Slovenia, and Malta) whose household internet access was within one standard deviation (6.50) from the European average (86.5%) as countries with a “medium” broadband internet usage; 11 countries (Bulgaria, Croatia, Israel, France, Greece, Latvia, Lithuania, Poland, Portugal, Romania, and Slovak Republic) whose household internet access showed more than a half standard deviation (3.25) from the European average as countries with a “low” broadband internet usage; and 10 countries (Cyprus, Denmark, Estonia, Finland, Germany, Luxembourg, the Netherlands, Spain, Sweden, and Switzerland) whose household internet access was at least half a standard deviation (3.25) above the European average as countries with a “high” broadband internet usage. This information refers to 2019 (Israel: 2018)—the year preceding the interview in 2020—and was drawn from the OECD (OECD, 2020b) and Eurostat (Eurostat, 2021) databases.

### Methods

**Figure 1:**
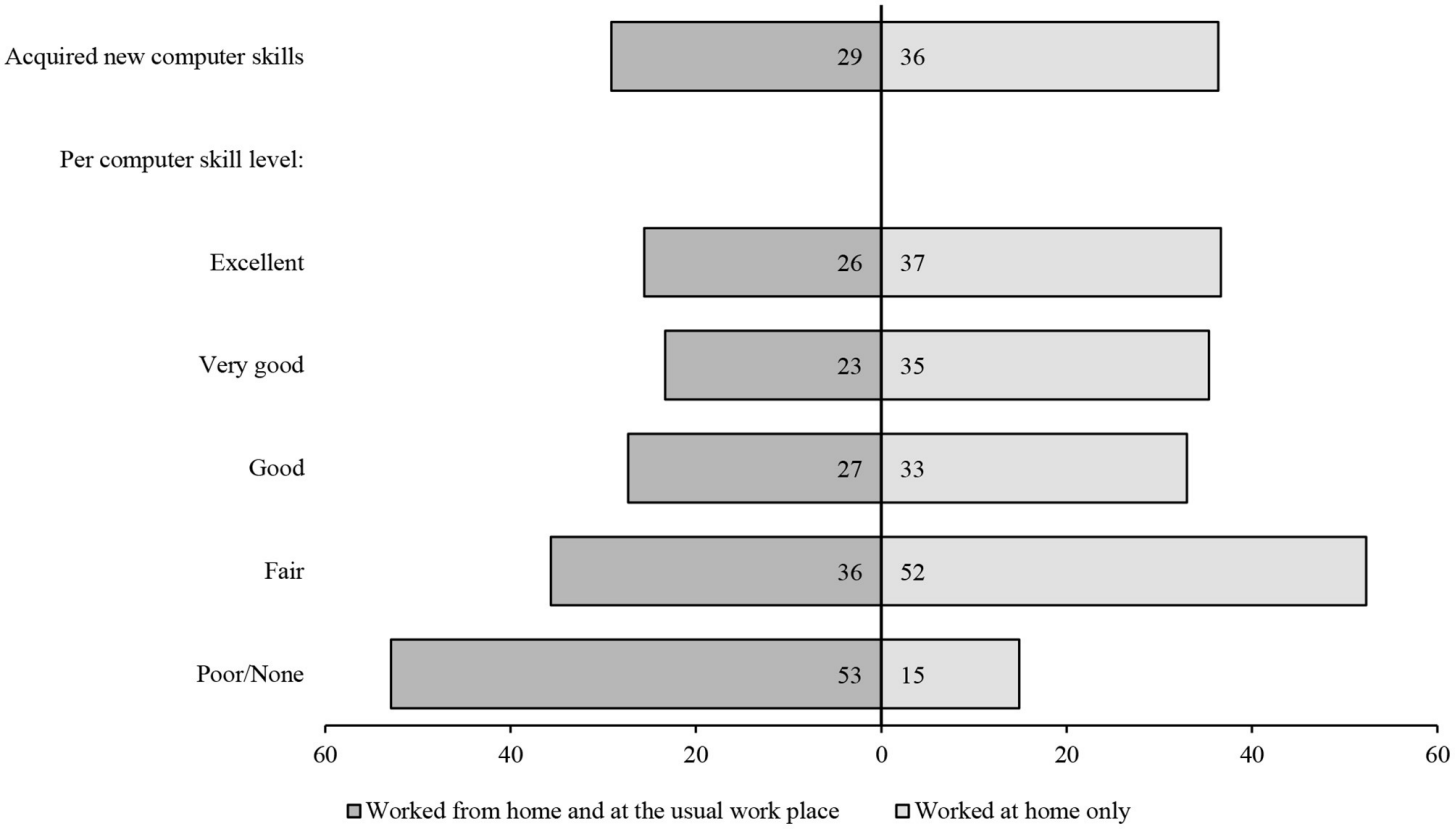
New Computer Skills due to Home-Office in Times of Covid-19 (Proportion). Presented are proportions. Authors' own graph. Data source: Survey of Health, Aging and Retirement in Europe (SHARE), wave 8, COVID-19 Survey 1, release 8.0.0; *N* = 3,197 (respondents w/o information on skill level are not shown); weighted, own calculations.

After providing a general overview of the main characteristics of employees aged 50 years and older across Europe (see Table 1), we answered our main question on the improvement in their computer skills due to homeworking related to COVID-19 (see Figure 1). Moreover, based on stepwise logistic regressions, we analyzed the relationship between homeworking and improved computer skills under the consideration of other relevant individual and structural influences (see Table 2). Furthermore, our multivariate analysis examined whether and which type of homeworking (partial or entire) might promote computer skills in older people as well as different patterns between these two types. Lastly, we considered interactions between the type of home working, workload, sex, education and national internet distribution to account for the complex mechanisms regarding the improvement in computer skills in later working life (see Table 3). All statistical analyses were conducted using Stata 16.0 (StataCorp, 2019).

**Table 2:** Determinants of improved computer skills.

	**Gross**			**M1**			**M2**			**M3**			**M4**			**M5**			**M6**			**M7-1**			**M7-2**		
	**OR**	**P**	**SE**	**OR**	**P**	**SE**	**OR**	**P**	**SE**	**OR**	**P**	**SE**	**OR**	**P**	**SE**	**OR**	**P**	**SE**	**OR**	**P**	**SE**	**OR**	**P**	**SE**	**OR**	**P**	**SE**
Worked at home only	**1.30**	0.000	0.10	**1.27**	0.001	0.09	**1.29**	0.001	0.10	**1.29**	0.001	0.10	**1.30**	0.001	0.10	**1.29**	0.001	0.10	**1.30**	0.001	0.10	**No**			**Yes**		
**Workload**																											
*Unchanged (Ref.)*																											
Reduced	1.05	0.550	0.09	1.15	0.124	0.11	1.16	0.114	0.11	1.15	0.136	0.11	1.14	0.161	0.11	**1.20**	0.065	0.12	**1.21**	0.050	0.12	1.11	0.480	0.17	**1.30**	0.046	0.17
Increased	**2.43**	0.000	0.24	**2.36**	0.000	0.24	**2.38**	0.000	0.24	**2.34**	0.000	0.24	**2.20**	0.000	0.22	**2.02**	0.000	0.21	**2.03**	0.000	0.21	**2.09**	0.000	0.33	**1.99**	0.000	0.28
Affected by unemployed, laid off or business closure	**0.60**	0.000	0.08	**0.62**	0.000	0.08	**0.62**	0.000	0.08	**0.62**	0.000	0.08	**0.58**	0.000	0.08	**0.60**	0.000	0.08	**0.61**	0.000	0.09	**0.60**	0.021	0.13	**0.61**	0.007	0.11
Internet connection	**0.77**	0.036	0.10	**0.81**	0.081	0.10	**0.81**	0.098	0.10	**0.80**	0.083	0.10	**0.80**	0.076	0.10	0.84	0.175	0.11	0.85	0.198	0.11	0.98	0.929	0.20	0.78	0.146	0.13
**Computer skills**																											
**Excellent** (**Ref.)**																											
Very good	**1.43**	0.004	0.18				**1.52**	0.001	0.19	**1.50**	0.001	0.19	**1.46**	0.003	0.19	**1.40**	0.010	0.18	**1.40**	0.011	0.18	1.28	0.224	0.26	**1.45**	0.033	0.26
Good	**1.55**	0.000	0.18				**1.63**	0.000	0.20	**1.62**	0.000	0.20	**1.57**	0.000	0.19	**1.56**	0.000	0.20	**1.55**	0.001	0.20	**1.38**	0.097	0.27	**1.65**	0.003	0.28
Fair	**1.47**	0.005	0.20				**1.57**	0.001	0.22	**1.55**	0.002	0.22	**1.54**	0.002	0.22	**1.65**	0.001	0.25	**1.64**	0.001	0.24	**1.48**	0.090	0.34	**1.75**	0.004	0.34
Poor/None	0.78	0.344	0.20				0.89	0.640	0.23	0.92	0.746	0.24	0.87	0.606	0.23	0.94	0.808	0.25	0.95	0.846	0.26	1.53	0.264	0.58	0.58	0.157	0.22
Missing (not asked yet)	**1.79**	0.000	0.28				**1.82**	0.000	0.29	**1.83**	0.000	0.29	**1.86**	0.000	0.30	**1.61**	0.005	0.27	**1.60**	0.006	0.27	**1.81**	0.020	0.46	1.39	0.155	0.32
Cognition score *(centered)*	**1.10**	0.000	0.03							**1.09**	0.001	0.03	**1.08**	0.005	0.03	1.01	0.802	0.03	1.01	0.823	0.03	0.99	0.862	0.04	1.01	0.697	0.04
**Pre-covid health**																											
***Excellent (Ref.)***																											
Very good	1.05	0.673	0.12							1.03	0.775	0.12	1.03	0.823	0.12	1.03	0.832	0.12	1.04	0.753	0.12	1.01	0.970	0.18	1.11	0.542	0.18
Good	1.11	0.325	0.12							1.09	0.423	0.12	1.07	0.550	0.12	1.12	0.311	0.13	1.13	0.280	0.13	1.27	0.161	0.22	1.05	0.764	0.17
Fair/Poor	1.13	0.392	0.16							1.07	0.643	0.15	1.03	0.857	0.15	1.10	0.518	0.16	1.10	0.524	0.17	1.09	0.720	0.25	1.15	0.495	0.23
Nervous																											
***No (Ref.)***																											
Yes	**1.49**	0.011	0.23										**1.39**	0.044	0.23	1.29	0.122	0.22	1.30	0.120	0.22	1.45	0.139	0.36	1.19	0.430	0.27
Yes & more since Covid	**1.52**	0.000	0.13										**1.44**	0.000	0.13	**1.28**	0.010	0.12	**1.28**	0.009	0.12	**1.46**	0.008	0.21	1.13	0.332	0.15
**Felt uplifted**																											
***No (Ref.)***																											
Yes, spontaneous	**1.64**	0.000	0.17										**1.57**	0.000	0.17	**1.50**	0.000	0.17	**1.50**	0.000	0.17	**1.44**	0.040	0.25	**1.55**	0.004	0.23
Yes, delayed	**1.36**	0.025	0.18										**1.28**	0.086	0.18	1.27	0.105	0.19	1.26	0.115	0.19	1.15	0.525	0.26	1.36	0.124	0.27
Looking forward																											
***No (Ref.)***																											
Yes, spontaneous	**1.82**	0.000	0.26										**1.51**	0.006	0.22	**1.56**	0.003	0.24	**1.57**	0.003	0.24	**1.78**	0.012	0.41	**1.40**	0.097	0.29
Yes, delayed	**1.88**	0.001	0.35										**1.67**	0.009	0.33	**1.74**	0.006	0.35	**1.76**	0.005	0.36	**2.10**	0.014	0.63	1.56	0.109	0.43
Female	**2.08**	0.000	0.16													**1.69**	0.000	0.14	**1.68**	0.000	0.14	**1.50**	0.001	0.18	**1.89**	0.000	0.22
Year of birth *(centered)*	**1.04**	0.000	0.01													**1.03**	0.000	0.01	**1.03**	0.000	0.01	**1.02**	0.084	0.01	**1.04**	0.000	0.01
Tertiary education	**2.17**	0.000	0.18													**2.05**	0.000	0.18	**2.03**	0.000	0.18	**2.03**	0.000	0.26	**2.05**	0.000	0.25
Migrant	**0.77**	0.066	0.11													0.82	0.181	0.13	0.81	0.156	0.12	0.99	0.949	0.23	**0.67**	0.056	0.14
Living alone	1.02	0.849	0.10													0.99	0.940	0.10	0.99	0.935	0.10	1.04	0.798	0.16	0.96	0.772	0.14
**National broadband access**																											
***Medium (Ref.)***																											
Low	**1.28**	0.014	0.13																1.10	0.372	0.12	1.08	0.640	0.18	1.14	0.378	0.17
High	**1.15**	0.100	0.10																1.15	0.136	0.11	**1.37**	0.029	0.20	1.02	0.903	0.13
N			3,197			3,197			3,197			3,197			3,197			3,197			3,197			1,473			1,724
Nagelkerke's *R*^2^						0.034			0.042			0.046			0.061			0.103			0.104			0.091			0.120
Model c-statistic						0.599			0.612			0.618			0.638			0.686			0.687			0.678			0.700

*Data sources: Survey of Health, Aging and Retirement in Europe (SHARE), wave 8, COVID-19 Survey 1, release 8.0.0, logistic regressions, odds ratios (OR), P value (P), robust standard errors (SE), references (Ref.), significant coefficients (p ≤ 0.100) are displayed bold, own calculations*.

**Table 3:** Interactions between determinants of improved computer skills.

**M8**		**OR**	**P**	**SE**		**M9**		**OR**	**P**	**SE**
**Worked at home only**	**Workload**					**National broadband access**	**Workload**			
*No (Ref.)*	*Unchanged (Ref.)*					*Medium (Ref.)*	*Unchanged (Ref.)*			
No	Reduced	1.11	0.469	0.16		Medium	Reduced	**1.72**	0.001	0.29
No	Increased	**2.06**	0.000	0.32		Medium	Increased	**1.90**	0.001	0.38
Yes	Unchanged	**1.26**	0.026	0.13		Low	Unchanged	1.22	0.186	0.19
Yes	Reduced	**1.63**	0.000	0.21		Low	Reduced	1.14	0.493	0.22
Yes	Increased	**2.54**	0.000	0.36		Low	Increased	**3.51**	0.000	0.76
						High	Unchanged	**1.32**	0.029	0.17
						High	Reduced	**1.46**	0.020	0.24
						High	Increased	**2.35**	0.000	0.40
*N*				3,197		*N*				3,197
Nagelkerke's R^2^				0.104		Nagelkerke's *R*^2^				0.107
Model c-statistic				0.687		Model c-statistic				0.690
**Worked at home only**	**Sex**					**National broadband access**	**Sex**		
***No (Ref.)***	***Male (Ref.)***					***Medium (Ref.)***	***Male (Ref.)***			
No	Female	**1.48**	0.001	0.17		Medium	Female	**1.95**	0.000	0.30
Yes	Male	1.12	0.357	0.14		Low	Male	**1.45**	0.040	0.26
Yes	Female	**2.12**	0.000	0.23		Low	Female	**1.85**	0.000	0.29
						High	Male	1.22	0.176	0.18
						High	Female	**2.14**	0.000	0.30
N				3,197		N				3,197
Nagelkerke's *R*^2^				0.104		Nagelkerke's *R*^2^				0.105
Model c-statistic				0.687		Model c-statistic				0.688
**Worked at home only**	**Tertiary education**					**National broadband access**	**Tertiary education**			
*No (Ref.)*	*No (Ref.)*					Medium (Ref.)	No (Ref.)			
No	Yes	**2.02**	0.000	0.26		Medium	Yes	**1.30**	0.086	0.20
Yes	No	**1.28**	0.084	0.19		Low	No	0.84	0.377	0.17
Yes	Yes	**2.63**	0.000	0.33		Low	Yes	**1.69**	0.001	0.26
						High	No	**0.69**	0.021	0.11
						High	Yes	**1.92**	0.000	0.26
*N*				3,197		N				3,197
Nagelkerke's *R*^2^				0.104		Nagelkerke's *R*^2^				0.108
Model c-statistic				0.687		Model c-statistic				0.691
**Worked at home only**	**National broadband access**					**National broadband access**	**Worked at home only**			
***No (Ref.)***		***Medium (Ref.)***				**Medium (Ref.)**	**No (Ref.)**			
No	Low	1.11	0.532	0.19		Medium	Yes	**1.50**	0.008	0.23
No	High	**1.36**	0.033	0.19		Low	No	1.11	0.532	0.19
Yes	Medium	**1.50**	0.008	0.23		Low	Yes	**1.68**	0.001	0.27
Yes	Low	**1.68**	0.001	0.27		High	No	**1.36**	0.033	0.19
Yes	High	**1.51**	0.004	0.22		High	Yes	**1.51**	0.004	0.22
*N*				3,197		*N*				3,197
Nagelkerke's *R*^2^				0.105		Nagelkerke's *R*^2^				0.105
Model c-statistic				0.688		Model c-statistic				0.688

*Data sources: Survey of Health, Aging and Retirement in Europe (SHARE), wave 8, COVID-19 Survey 1, release 8.0.0, separate logistic regressions (models under control of all variables included in M6 of Table 2), odds ratios (OR), P value (P), robust standard errors (SE), references (Ref.), significant coefficients (p ≤ 0.100) are displayed bold, own calculations*.

## Results

### Working at Home During the Pandemic and New Computer Skills: A Descriptive Overview

A first glance (see Table 1) at the data from our 11,042 employed respondents aged 50 years and older revealed that 12% said they worked at home only due to the pandemic, while 15% alternately worked from home and at their usual workplace. However, more than one in two of the respondents (54%) continued to work at their usual workplace, while 19% said that such working arrangements did not apply to them. These 19% were those whose employment was directly affected by the pandemic, and most of whom could not continue their work either at their usual workplace or at home. Overall, 53% of all respondents who were faced with unemployment, being laid off, or business closures belonged to this group of employees.

Although the type of continued employment during the pandemic did not differ greatly according to sex, cohort, migration history, and living situation, there were clear variations with regard to educational background and preexisting computer skills. The descriptives point out that those with no tertiary educational attainment seemed to be doing more jobs that could not be done from home (see Table 1). Whereas, 21% of the respondents who continued to work in their usual working environment had tertiary educational credentials, this proportion was almost three times higher among people who were partially or entirely working from home (57 and 62%, respectively). A similar picture emerged for the respondents' individual computer skills. Around 40% of the part-time and full-time homeworkers rated their computer skills as “Excellent” or “Very good,” whereas only 17% of those who kept working in their regular working environment claimed the same. Regarding the differences between those who worked at home full time and those who did so only part time, the descriptives indicate that full-time homeworking was more of an option or was more common among those with a higher educational attainment and who already had excellent computer skills.

The main aim of this study is to investigate whether and how the computer skills of full-time and part-time homeworkers changed during the pandemic with their new working environment. Overall, the majority of the homeworkers did not report that they had acquired new computer skills. However, the respondents who worked entirely from home reported a higher extent of newly learned computer skills than those who worked only part time from home (Figure 1). More precisely, 36% of the older employees who worked only at home reported an increase in their computer skills, whereas only 29% of the older workers who worked alternately at home and at their usual workplaces reported such an improvement. The older workers who were working entirely at home and who had rated their previous computer skills as “Fair” showed the highest gain (52%) in IT knowledge. Interestingly, the small proportion of older workers who were working part time at home and who had reported “Poor” or even no IT knowledge (2%) showed the highest gain (53%) in IT knowledge. In summary, the results highlight that a considerable proportion of the respondents were able to improve their computer skills regardless of their previous knowledge.

### Determinants of Gaining New Computer Skills

The previous results indicate that working from home (entirely and partially) improves the computer skills of a substantial percentage of older employees. To test which type of homeworking (entire or partial) is likelier to improve computer skills in later working life, and to explain which other factors and circumstances lead to improved IT knowledge, we performed stepwise logistic regressions (Table 2). Prior to our multivariate modeling, we first measured the gross effect of each independent variable and further tested for multicollinearity, whereby the variance inflation factor (VIF) of all the individual variables did not exceed 10.0 (mean 2.57). Further, both non-dichotomous independent variables (cognition score and year of birth) were included as mean-centered items in our empirical setting.

In the first step (M1), we included work-relevant factors in the analysis. The findings show that the complete switch to the home office significantly improved the computer skills of older workers. These older workers were also precisely those respondents whose working hours increased on average in the wake of the pandemic and who were less affected by work interruptions, such as unemployment, layoffs, or even business closures. Interestingly, adequate internet speed at home was not necessary for improved computer literacy. This suggests that those with a high private internet speed already had better computer skills even before the pandemic. By including such previously acquired computer skills in our analysis (M2), the results showed that the home office led to a significant improvement in the computer skills of older people with fair, good, or very good IT knowledge. By contrast, those who previously rated their digital skills as “Excellent” or “Poor” (including those who indicated that they had no computer skills) did not learn new computer skills from working at home.

With regard to the link between pre-COVID-19 cognition and health and the improvement in computer skills (M3), workers with higher cognitive skills seemed to be more able to acquire new knowledge and thus were more likely to report improved computer skills. However, the state of health had no impact on this skill acquisition. Since COVID-19 has affected the lives of almost everyone and in various dimensions, we further tested the personal traits related to the pandemic and their influence on newly acquired computer skills (M4). The results showed two seemingly contradictory results. First, respondents who recently felt (increasingly) nervous, anxious, or on edge were likelier to gain new computer skills during this phase of the pandemic in comparison to those who had not such feelings. Second, we found the same pattern for a gain in computer skills for employees who had uplifting experiences during the outbreak that gave them inspiration or happiness, and those who said they were looking forward to the time when COVID-19 would abate in contrast to respondents who did not report such positive experiences or expectations.

Model 5 controlled for the respondents' demographics and living conditions. It showed that women in particular experienced improved computer skills from working from home. The younger among the older generation reported the same. Further, those with tertiary education degrees were also more likely to report improved computer skills during the pandemic. We found no influence of migration or the current living situation on changed computer skills. The respondents' years of birth and sex influenced their self-reported improvement in computer skills in two ways: with a loss and with a gain. In line with previous findings (see for example, Foverskov et al., 2018; Kamin and Lang, 2020), this can be attributed to the natural decline in cognitive abilities with age and to the fact that the women under investigation achieved—despite a similar age distribution—higher cognition scores than men (t-test significant at the *p* = 0.001 level). However, employees whose working hours were reduced due to the pandemic were now also more likely to report that they had acquired new computer skills. This pattern applies mainly to younger employees with tertiary qualifications.

Model 6 considered the country-specific distribution of broadband internet access. Whereas, the gross effect indicated improved computer skills for respondents living in countries with national broadband access below and above the European average, the analysis that covered all relevant indicators underlined that the direction of this pattern remained true but simultaneously lost its significance.

Based on these findings and to investigate different mechanisms depending on the type of homeworking experienced, we tested the model specified under M6 separately for employees who worked partially (M7-1) or entirely (M7-2) at home. Overall, the results confirmed the previously found mechanisms and pointed out that women, younger employees, those who are highly educated, and those whose work was not affected by unemployment, layoffs, or even business closures acquired new computer skills significantly more often, regardless of whether they worked entirely or partly from home.

However, we simultaneously found differences depending on the type and extent of homeworking. Partial homeworking respondents who increased their working time as a result of the pandemic as well as those, who showed increased feelings of nervousness or anxiety were likelier to have learned new computer skills since the outbreak. The same outcome was observed with respondents, who worked partially at home and who resided in one of the ten countries with a generally higher internet distribution such as Cyprus, Denmark, Estonia, Finland, Germany, Luxembourg, the Netherlands, Spain, Sweden, and Switzerland. A similar increase in computer knowledge was seen among full-time homeworkers, especially those whose regular working hours had to be either reduced or increased, those who already had in-depth IT skills before the pandemic, and those who had no prior migration experiences and thus belonged to the native population. The results further showed no influence from the national internet infrastructure on the acquisition of new computer skills for those who were working full time at home.

Based on these findings and to capture a broader picture of the effects of the type of homeworking (M8) and national broadband access (M9) on improved computer skills, we included several interaction terms for changed workload, educational background, and sex as indicators for each combination of the categories of the variables used in the equation (Table 3).

Here, the results revealed that respondents who had worked entirely at home since the outbreak of COVID-19 were more likely to report an improvement in their computer skills. The same applies to partial homeworkers who increased their workload since the spread of coronavirus. A similar effect could be found regarding the interaction between national broadband access and workload for those from countries with an overall better ICT infrastructure. However, respondents from Bulgaria, Croatia, Israel, France, Greece, Latvia, Lithuania, Poland, Portugal, Romania, and Slovak Republic who lived in countries with a below average distribution of broadband access but who increased their regular working hours could also benefit from improved computer skills. The same applies to employees from countries with “medium” national broadband access (Austria, Belgium, Czech Republic, Hungary, Italy, Slovenia, and Malta) who either increased or reduced their workload.

Regarding sex-specific differences, the included interactions highlight that women in general were more able to improve their computer skills since the outbreak of the pandemic, regardless of their working situation (partial or entirely at home) and country of residence. Among men, only those from countries with below-average ICT infrastructure reported an improvement in their computer knowledge.

A similar picture was observed for highly educated employees. In comparison to respondents without tertiary qualifications, those with tertiary credentials reported improved computer skills significantly more often, regardless of their working environment (entirely or partially at home) or country. However, for respondents with non-tertiary education, the results indicate that their computer skills also improved if they worked entirely at home. Simultaneously, employees with non-tertiary education but living in countries with “high” national broadband access were significantly less likely to increase their computer skills during this phase of the pandemic.

Finally, the interaction between the type of homeworking (partially or entirely) and country-specific internet access showed that, in general, respondents who were permanently working at home significantly more often improved their computer skills, regardless of the ICT infrastructure in their country. Only in countries with a comparatively above-average availability of high-speed internet does the type of homeworking play no role in learning new skills in later life.

## Discussion

Our analysis showed that only 12% of the respondents worked full time at home due to the COVID-19 restrictions, while 15% worked partly from home and partly at their usual workplace. The descriptives indicate that full-time homeworking was more common among those with tertiary education and who already had excellent computer skills before the pandemic. Therefore, working from home was not an option for each older worker, which underlines the possible social inequality of telework (Sostero et al., 2020).

Overall, the majority of the older workers who were working from home did not report acquiring new computer skills. However, more of those who worked full time from home reported that they acquired new computer skills than those who worked only partly from home. This reveals a certain “digital push” among the older workers who were working from home (Gallistl et al., 2021). Such a digital push suggests an increase in digital use and skills due to COVID-19 home-related use of ICT (e.g., the internet) for work or social interactions (e.g., video calls with family members). Amankwah-Amoah et al. (2021) also described COVID-19 as “the great accelerator,” having fast-tracked the existing global trend toward embracing modern technologies and having ushered in transformations in work life (Amankwah-Amoah et al., 2021).

Against the background of the concept of “digital capital” (Park, 2017; Ragnedda, 2018), our analyses highlight an increase in digital capital, in this case, in computer skills, to some extent related to homeworking due to COVID-19. These new computer skills can be understood as new resources for the digitally oriented future work of older workers aged 50 years and older, and thus as enriching their digital capital set, which will open up to them better opportunities in the labor market even after the COVID-19 pandemic (Milasi et al., 2021).

Nevertheless, not all older workers reported an increase in computer skills during COVID-19-related homeworking. In fact, the results highlight that those with few or some digital experiences especially benefited from improved computer skills through pandemic-related homeworking compared to older adults with already excellent computer skills before the pandemic.

Overall, the results point out that women, younger respondents, employees with tertiary education, and those whose work was not affected by unemployment, layoffs, or even business closure acquired new computer skills significantly more often, regardless of whether they worked entirely or partly from home. The results further showed that those who worked only partly at home were less able to improve their computer skills than those who switched completely to work from home. Moreover, the previously recurring difference between men and women in the observed age group in terms of technology use (König and Seifert, 2020) was apparently narrowed with the pandemic, as women showed a higher increase in computer skills during the pandemic and regardless of whether they worked entirely or partially from home and regardless of their country of residence. Therefore, the pandemic can be seen as a push factor in acquiring computer skills in later working life, especially for women. However, to overcome the digital gap among older workers, it is important to invest in training to increase digital capital, even after the pandemic (Cros et al., 2021). This also includes a further expansion of the internet infrastructure to guarantee adequate use for everyone—even beyond a pandemic.

Even if working from home is not possible for some older workers, the current pandemic has shown that new computer skills are important in maintaining employment remotely and even non-remotely in old age, within the current scenario, but even more so in the future, in which everyone's work is expected to be digitally dominated. Against the expected accelerated development of new technologies, older workers can remain employed and be empowered to cope with new work-related digital challenges if they are able to use such technologies. Getting older adults to use ICT is one challenge, but once they are already using, for example, work-related new software, getting them to continue using other ICTs is another challenge. Maintaining the use of digital devices by older adults is an understudied but critical area for future research. When ICTs stop working properly, need repairs, require software, and password updates, and change their layout and appearance through software interface updates, some older adults may not be able to keep using them over time (Houston et al., 2019). During such times, older adults may need assistance from IT professionals in their workplace or from their social ties to resolve IT issues and continue using the technology (Kamin et al., 2020). If these sources of support are not available, older adults may end up using the technology less frequently or not at all (König and Seifert, 2020). Although the current pandemic motivated older workers to learn new computer skills, there is a need for targeted funding and programs to meet the needs of these individuals to remain connected to the labor market. This is vital because nearly all occupations are increasingly requiring digital skills, including those that have not traditionally required them, and remote work is becoming more common (Hecker et al., 2021).

As people aged 50–60 years grow older, it will be interesting to see how their technology use evolves as new ICTs continue to be developed and disseminated and whether they will be able to maintain their usage of various types of work-related ICT. Technology is constantly evolving; the fast-paced nature of technology development leaves researchers with the challenge of staying abreast of the latest developments in technology and how its use is evolving over time. This also contributes to the limited understanding of the impacts of technology use on older adults and how to maintain their use of such technology over time. Interdisciplinary research, as well as cooperative studies between technology developers and researchers in the sociology and business domains, will be needed in the future to more fully comprehend the many ways in which older adults use ICT, how their ICT use waxes and wanes over time, and the complex pathways through which ICT use may have an impact on older adults' working lives, even after the COVID-19 pandemic.

### Limitations

As this study focused on Europe, our findings may have limited generalizability outside of Europe. Moreover, our dependent variable was limited (i.e., there was no information on areas, forms, and levels of computer skills), as not all possible facets of digital skills gained during the pandemic could be considered. Hence, future studies are needed to measure in greater detail the computer skills (objectively and subjectively) of older workers in their home offices and the changes or stability of such skills over time.

Although the SHARE data allowed us to investigate an improvement in digital skills in a variety of European countries, especially among older adults, the information is limited to employees whose profession and employer allowed them to switch to a home office in the wake of the pandemic. In addition to this limitation to a certain part of the workforce, the survey also lacked several important variables, such as technology biographies, attitudes toward technology, technology acceptance, the use of technology in the household, and reasons for non-use. Moreover, the SHARE dataset used does not allow the differentiation between work locations and their technical frameworks, requirements (e.g., required employee skills), or in-house computer training provided. Furthermore, we do not know about the respondents' use of continuing education services or tools to build their digital skills during the pandemic. Future studies with representative data should focus on longitudinal settings to investigate more deeply the factors that influence the acquisition of digital skills and the educational needs of older adults in later working life.

## Conclusion

For the European countries included in this study, our findings revealed a considerable pandemic-related increase in computer skills among workers aged 50 years and older while working from home due to the worldwide COVID-19 pandemic. Such an increase in computer skills was observed, especially for women and among people who already had some computer skills before the pandemic. Furthermore, the increases in computer skills were driven by socioeconomic and work-related conditions. Even though the COVID-19 pandemic has, to some extent, increased the “digital capital” of older (female) employees, work-related interventions (such as computer skills training among older workers) should be pursued to further promote the digital skills of workers to prepare them for remote work and, generally, for the digital transformation of work in the future.

## Data Availability Statement

Publicly available datasets were analyzed in this study. This data can be found here: http://www.share-project.org/.

## Author Contributions

All authors listed made substantial, direct, and intellectual contributions to the work and approved it for publication.

## Funding

The SHARE data collection has been funded by the European Commission, DG RTD through FP5 (QLK6-CT-2001-00360), FP6 (SHARE-I3: RII-CT-2006-062193, COMPARE: CIT5-CT-2005-028857, SHARELIFE: CIT4-CT-2006-028812), FP7 (SHARE-PREP: GA N°211909, SHARE-LEAP: GA N°227822, SHARE M4: GA N°261982, DASISH: GA N°283646) and Horizon 2020 (SHARE-DEV3: GA N°676536, SHARE-COHESION: GA N°870628, SERISS: GA N°654221, SSHOC: GA N°823782, SHARE-COVID19: GA N°101015924) and by DG Employment, Social Affairs & Inclusion through VS 2015/0195, VS 2016/0135, VS 2018/0285, VS 2019/0332, and VS 2020/0313. Additional funding from the German Ministry of Education and Research, the Max Planck Society for the Advancement of Science, the U.S. National Institute on Aging (U01_AG09740-13S2, P01_AG005842, P01_AG08291, P30_AG12815, R21_AG025169, Y1-AG-4553-01, IAG_BSR06-11, OGHA_04-064, HHSN271201300071C, RAG052527A) and from various national funding sources is gratefully acknowledged (see www.share-project.org). The data were adjusted for this investigation, for which extensive consistency checks were made.

## Conflict of Interest

The authors declare that the research was conducted in the absence of any commercial or financial relationships that could be construed as a potential conflict of interest.

## Publisher's Note

All claims expressed in this article are solely those of the authors and do not necessarily represent those of their affiliated organizations, or those of the publisher, the editors and the reviewers. Any product that may be evaluated in this article, or claim that may be made by its manufacturer, is not guaranteed or endorsed by the publisher.
